# Development of a Balance Recovery Performance Measure for Gait Perturbation Training Based on the Center of Pressure

**DOI:** 10.3389/fspor.2021.617430

**Published:** 2021-02-15

**Authors:** Markus M. Rieger, Selma Papegaaij, Frans Steenbrink, Mirjam Pijnappels, Jaap H. van Dieën

**Affiliations:** ^1^Department of Human Movement Sciences, Amsterdam Movement Sciences, Vrije Universiteit Amsterdam, Amsterdam, Netherlands; ^2^Motek Medical BV, Amsterdam, Netherlands

**Keywords:** postural balance [MeSH], walking, gait, accidental falls, physical functional performance, rehabilitation, aging

## Abstract

**Background:** The availability of instrumented treadmills that can apply unexpected perturbations during walking has made gait perturbation training more popular in clinical practice. To quantify and monitor balance recovery while training, easy to use measures are needed and may be based on integrated force plate data. Therefore, we aimed to quantify and evaluate different implementations of the recovery performance measure based on center of pressure data.

**Methods:** Recovery performance was calculated based on differences in center of pressure trajectories between unperturbed walking and balance recovery after a perturbation. Five methodological choices leading to 36 different implementations were evaluated. Test-retest reliability, effect sizes, and concurrent validity were evaluated against trunk velocity measures.

**Results:** Differences in measures of (dis-)similarity, time normalization and reference data affected reliability, sensitivity and validity and none of the performance measure implementations based on center of pressure trajectories was superior on all criteria. Measures assessing perturbation effects on trunk velocities provided more reliable and sensitive recovery outcomes.

**Discussion:** Different implementations of the recovery performance measure can be chosen dependent on constraints imposed in the clinical setting.

**Conclusion:** Quantifying recovery performance based on center of pressure data is possible and may be suitable to monitor improvement in recovery performance after gait perturbations in specific clinical setups. Validity of performance measures in general requires further attention.

## Introduction

Fall prevention training using gait perturbations during walking is becoming more popular (Gerards et al., 2017), but the application of standardized and sufficiently impactful perturbations is generally limited to setups that require a lot of space and are expensive and complex to control (e.g., over ground walkways or gait labs). Smaller devices, among which treadmills, are being developed to make gait perturbation training accessible in clinical settings. One advantage of advanced research setups is the ability to capture movements and record forces, to quantify the unperturbed gait kinematics and kinetics, the perturbation magnitude and impact, as well as the patient's balance recovery performance. Quantification of recovery performance is key for successful clinical application. It may also allow for identification of people at higher risk for falling and indicate necessity of fall-preventive interventions. Furthermore, it allows standardization and monitoring of the patient's progress over training sessions. This enables therapists to consistently adjust perturbation difficulty, to keep the patient challenged and motivated, and may support reporting outcomes to health care providers.

When loosing balance due to gait perturbations, the neuromotor system applies various strategies to regain balance (Hof et al., 2010; Reimann et al., 2018; van den Bogaart et al., 2020). According to Hof (2007), this can be achieved by adjusting the position of the center of pressure (CoP) relative to the vertical projection of the center of mass (CoM), by counter rotations of body segments around the CoM or by applying external forces (e.g., holding on to a handrail). Furthermore, older adults show compensatory stepping reactions (Jensen et al., 2001) and often take multiple steps in response to both anterior-posterior and medio-lateral perturbations (Mille et al., 2013).

In literature, recovery performance has been quantified in various manners. One commonly used parameter is called the margin of stability, which relates the movement of the center of mass (CoM) to foot placement (Hof et al., 2005). Although this measure is straightforward to use in unperturbed walking, its use is limited for large perturbations, due to the wide variability in balance recovery responses in terms of stepping direction, skipping instead of stepping, number of steps used, and use of other strategies than adjusting the position of the CoP, such as speeding up or slowing down (Bruijn et al., 2013; Hak et al., 2013). Consequently, it is not easy to infer how changes in the margin of stability contribute to recovery.

Maybe a more suitable approach is to quantify recovery performance after perturbations based on trunk kinematics (Owings et al., 2001; Grabiner et al., 2008; Bruijn et al., 2010; Sessoms et al., 2014; Roeles, 2018), as the trunk has a large impact on balance, given its large mass and cranial location. Trunk flexion angle at toe-off and trunk flexion velocity at recovery foot contact have been related to the successful balance recovery (van den Bogert et al., 2002; Sessoms et al., 2014). By combining linear and angular trunk velocities, the whole recovery movement of the trunk can be captured and compared to the trunk movements during normal walking. The deviation from normal walking can therefore be used to describe the perturbation impact and the rate of return to normal walking (Bruijn et al., 2010; Roeles, 2018; Rieger et al., 2020). The advantage of this approach is that it is less sensitive to variability in reactive stepping strategies.

While inertial measurements may allow low-cost motion capture compared to optical systems, at present, motion capture is often not available in clinical practice, so the measures mentioned above cannot be used. A simple solution, which would be more accessible (i.e., less time consuming), is the use of treadmills with embedded force plates. The cheapest option here is a one-directional force plate, which only records vertical ground reaction forces, and may provide sufficient information to quantify recovery after gait perturbations. To our knowledge, measures based on force plates to quantify balance recovery during gait have not been investigated previously. Finally, for successful clinical application, recovery performance should be quantified as single value, that is easy to interpret and available online during training, immediately after each perturbation.

Taking these constraints into account, the purpose of the study was to develop several implementations of a new potential measure of what we coined quantified recovery performance (QRP). These implementations all compare the CoP trajectory during balance recovery from a perturbation, but with small differences in data processing. We evaluated test-retest reliability, sensitivity and concurrent validity of these different measures against motion-captured based measures of trunk velocity. We hypothesized that the QRP has sufficient reliability, validity, and sensitivity to change to be used to monitor progress in fall-prevention training.

## Materials and Methods

### Participants

Data of a previous perturbation intervention trial were used for this study (Rieger et al., 2020). The cohort consisted of 30 healthy older adults aged 65 years or older, who had no experience with perturbation training. Any neurological, cardiovascular or pulmonary comorbidity (i.e., stroke, heart attack, hypertension) that occurred in the past 12 months, as well as orthopedic complications (i.e., lower extremity fractures, joint replacements) within the past 6 months before the study, led to exclusion.

### Experimental Setup

The setup and perturbation characteristics are explained in detail in the original study (Rieger et al., 2020). Briefly, participants walked on the GRAIL (Gait Real-time Analysis Interactive Lab, Motek Medical BV, Amsterdam, The Netherlands), a 3D instrumented dual-belt treadmill, with an integrated motion capture system (Vicon Motion Systems Ltd, Yarnton, UK). A model [Human Body Model (HBM), version 2.0, Motek Medical BV, Amsterdam, The Netherlands] based on 26 reflective markers placed on the feet, legs and trunk was used to capture the participants' movements.

A custom application (D-flow version 3.30.1, Motek Medical BV, Amsterdam, The Netherlands) triggered perturbations at heel strike (Zeni et al., 2008) of either the left or right leg while walking with a fixed treadmill speed at 1 m/s. ML perturbations consisted of a sideways movement of the treadmill platform to the side opposite of the foot contact, provoking cross-over stepping. AP perturbations consisted of belt decelerations at foot contact, provoking backwards balance loss. Participants were measured three times in total (Rieger et al., 2020). On the first day, their gait was perturbed eight times (four times in AP and four times in ML direction) before and after a short intervention consisting of 8 min of treadmill walking with 16 AP perturbations (experimental group) or without (control group). After a 1-week retention period, participants were measured again with the same eight perturbations. Results indicated, that short exposure to gait perturbations led to significant improvements in balance recovery (stabilization of the trunk during walking) that were retained over 1 week, which limits conclusion if training effects transfer between perturbation directions. Steady state gait parameters did not change compared to baseline, so we can conclude that improvements are based on improved reactive responses. Balance recovery was quantified based on trunk kinematics and we used this as a reference measure for testing concurrent validity in the current study. A detailed description can be found elsewhere (Rieger et al., 2020). In short, time series of trunk velocities of unperturbed and perturbed walking were normalized to 101 samples per stride. For unperturbed gait, averages over 100 strides and their variability for each percentage of the gait cycle were calculated. Deviations in perturbed gait relative to unperturbed gait were calculated for six degrees of freedom (Bruijn et al., 2010). Next, the deviations were divided by the standard deviation of the unperturbed gait cycle for each dimension and then combined as the Euclidian sum over degrees of freedom into a trunk velocity deviation measure (Bruijn et al., 2010). The integral of the deviation over the first three recovery strides following a perturbation, expressed as the area under the curve (AUC), was used to describe recovery performance for every perturbation. Initial exposure to perturbations caused an improvement in recovery performance in both groups. Hence, we used the pre- and post-intervention trials of the whole cohort to assess sensitivity. No change in recovery performance was found in the control group between the post-intervention and retention measurements. Hence, the post-intervention and retention trial of the control group were used to assess test-retest reliability. Finally, to assess concurrent validity we used the retention trial over the whole cohort, excluding the pre- and post-intervention trials that would add repeated (dependent) measurements.

### Data Processing

Trunk marker data and force plate data, recorded at baseline, post-intervention and retention, were processed with Nexus software (version 2.7.0, Vicon Motion Systems Ltd, Yarnton, UK) and custom MATLAB scripts (version R2018a; MathWorks Inc, Natick, MA, USA). Three-dimensional marker data were smoothed using a second order 15 Hz low-pass Butterworth filter. Deviation in linear and angular trunk velocities from normal walking were used to quantify performance during balance recovery following a perturbation (Rieger et al., 2020). Vertical force and moment data of the instrumented dual-belt treadmill, recorded at 1,000 samples/s, were combined to simulate a single force plate and to estimate the CoP time series, which were then smoothed with a second order 6 Hz low-pass Butterworth filter and a second order 0.5 Hz high-pass Butterworth filter to correct for drift in the position on the treadmill. Finally, CoP data were resampled to 100 samples/s to match 3D marker data.

### Quantified Recovery Performance

**Figure 1:**
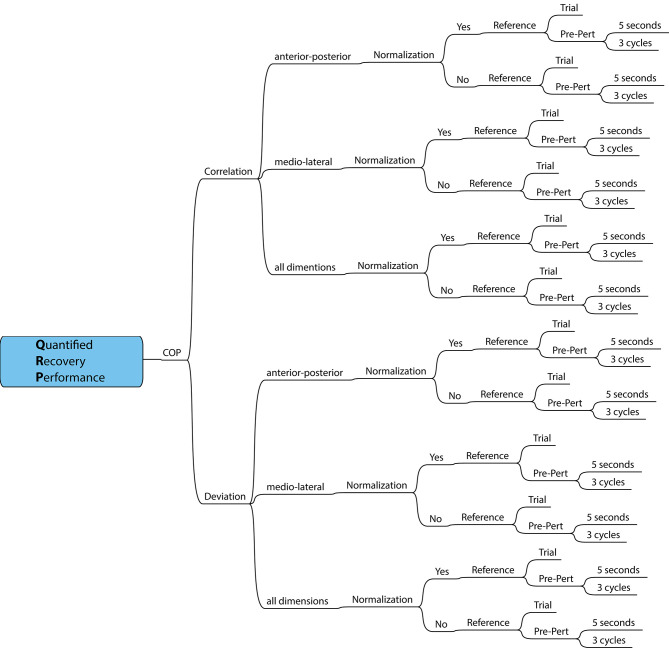
Schematic overview of methodological choices in data processing, resulting in 36 QRP measures.

The QRP is based on the fact that humans have a relatively constant gait pattern during unperturbed walking with the movements in each gait cycle being approximately the same. This gait pattern also results in a relatively constant CoP trajectory. The CoP is the point of application of the ground reaction force vector. This single point on the supporting surface is an effect of the forces that the individual exerts on the surface during walking. The proposed QRP utilizes this property of gait, since any perturbation will result in a change from this pattern. The QRP was calculated in different ways based on five methodological choices in data processing, with two to three options each, leading to 36 different implementations (Figure 1). Here, we describe the different choices that were made in the algorithm.

(1) The change from the normal walking pattern was quantified using Pearson's correlation coefficient or using an area under the curve describing the difference in the time series of the CoP patterns between perturbed and unperturbed gait as a measure of deviation.

(2) The next choice considered which CoP dimension to use. A pilot study suggested that change from the normal walking pattern was larger in AP compared to ML direction, when perturbations were applied in AP direction and vice versa for perturbations in ML direction. To cover the whole recovery reaction, the two directions can be combined. For the correlation-based calculation AP and ML coefficients were averaged using the Fisher z-transformation before averaging to avoid interpretation bias if the sampling distribution of correlation values is skewed, followed by the inverse transformation. For deviation-based calculation, both dimensions are combined as the Euclidian sum over dimensions.

(3) For perturbed gait, time normalizing the gait cycles may result in an average gait cycle that may be stretched unnaturally due to missed gait events when using an automatic gait event detection algorithm. One the other hand, because absolute step times may be different after a perturbation, time normalization may improve the comparison between unperturbed and perturbed gait episodes.

(4) To obtain an unperturbed gait pattern as reference, either a short episode of pre-perturbed walking preceding a perturbation or a separate trial of unperturbed walking can be used. The latter would allow a more reliable estimate as many gait cycles can be measured, but this comes at the cost of a longer measurement time. In our study, we recorded a 2-min steady state walking trial at 1 m/s. A template of unperturbed walking is then created by repeating the average gait cycle of these different references.

(5) Finally, the short episode of pre-perturbed walking was determined either by a number of gait cycles or a number of seconds. A measure dependent on the number of recovery steps requires accurate gait event detection. If not robust enough, manual post-processing is needed to evaluate whether events are correct or missing. The variance of recovery strategies and time to recover normal walking is large between participants. In a pilot study with older adults, therapists were not able to visually observe any effect of a perturbation after 5 s and our previous study showed that three cycles are enough for recovery of normal walking based on changes in trunk velocities (Rieger et al., 2020). The length of the post-perturbed walking episode was equivalent to that of the pre-perturbation episode.

### Example Calculation of a QRP Implementation

**Figure 2:**
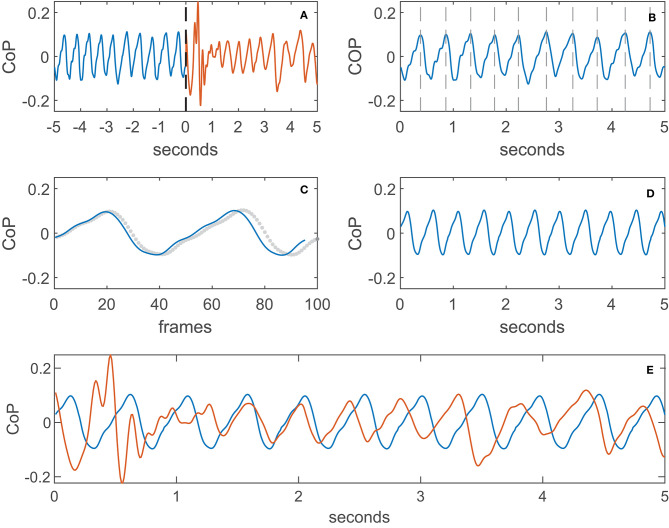
Example calculation of the QRP, **(A)**: pre- and post-perturbation window (black dashed line is the trigger), **(B)**: divided into gait cycles, **(C)**: de-normalization of the gait cycle (dotted line is the normalized gait cycle), **(D)**: template of unperturbed walking (averaged), **(E)**: alignment of template (averaged) and perturbed (non-normalized) walking signal.

The section above introduced the different choices that can be made within the algorithm. Here we describe, as an example, the details of the QRP calculation using the CoP trajectory in the AP dimension with a non-normalized time window of 5 s before and after the perturbation trigger, which are then compared using Pearson's correlation:

Step 1: The last 5 s before and the first 5 s after the perturbation of the CoP trajectory are selected and stored as a pre- and post-perturbation window (Figure 2A).
Step 2: In pre-perturbed CoP data, gait events are detected according to the method of Zeni and colleagues (Zeni et al., 2008). The gait cycles of the pre-perturbed episode are determined by the right or left heel strikes as the start and end of a gait cycle (Figure 2B).
Step 3: The average length of the gait cycles is calculated and the gait cycles of the pre-perturbed episode are time normalized to this average gait cycle length. In case of a non-normalized pre-perturbed episode, the gait cycle is de-normalized again after averaging (Figure 2C). For unperturbed walking, the average gait cycle is repeated multiple times to construct a template with reduced variance (Figure 2D).
Step 4: The cross-correlation between the post-perturbation CoP trajectory and the constructed unperturbed walking template is used to align the two signals (Figure 2E). The maximum correlation is used as a measure of recovery performance. In case of AUC, the template (unperturbed episode) is cut to the length of the perturbed episode.

### Statistics

Statistical analyses were performed with SPSS version 25 (SPSS Inc, Chicago, IL, USA). First, we checked for normality of data using the Shapiro-Wilk test. Second, the interquartile range rule (IQR) was used to detect outliers, with no extreme outliers (exceeding three times IQR) being found and consequently no observations were excluded from the analysis. The level of significance was set at alpha = 0.05.

To evaluate between session test-retest reliability we calculated parametric intraclass correlation coefficients (ICC) with two-way mixed single measure analyses for consistency between the post-intervention and the retention trials of the control group. ICCs were interpreted according to Shrout (1998) as indicating insufficient reliability (<0.40), fair reliability (0.40–0.60), moderate reliability (0.60–0.80), and substantial reliability (>0.80). Next, sensitivity to change was assessed by the effect size (ES) calculated with the mean difference over time divided by the standard deviation of the difference for the pre- and post-intervention trials of the whole cohort. An ES of 0.2 and lower reflects a mean difference of two measurements of <0.2 standard deviations, which can be interpreted as a trivial effect, even if results are significant. An ES between 0.5 and 0.8 is considered as a medium effect and above 0.8 as a large effect (Cohen, 1992). Finally, concurrent validity of the retention trial was tested with Pearson's correlation coefficient between the QRP and recovery performance based on trunk velocity deviation (Rieger et al., 2020). Pearson's r were interpreted as weak (below *r* = 0.3), moderate (*r* = 0.31–0.69), and strong (above *r* = 0.7).

## Results

**Figure 3:**
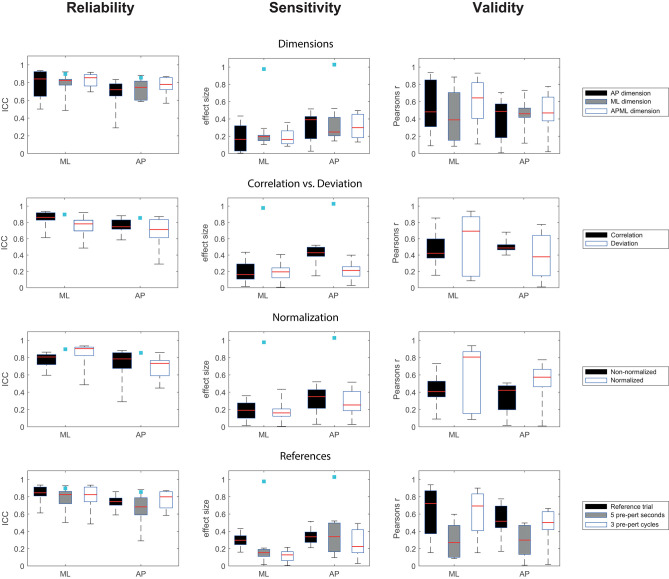
Boxplots of outcomes for different methodological choices of QRP implementations, x-axis: ML, medio-lateral perturbation; AP, anterior-posterior perturbation, light blue squares indicate the results of the trunk velocity deviation measure.

Measures of trunk velocity deviation resulted in substantial between session reliability of ICC = 0.897 for ML perturbations and ICC = 0.855 for AP perturbations (Figure 3). The sensitivity to change was ES = 0.977 for ML perturbations and ES = 1.028 for AP perturbations which is considered to be a large effect.

For the QRP measures, between session reliability ranged from fair to substantial for ML perturbations with ICC = 0.486–0.935 and from insufficient to substantial for AP perturbations with ICC = 0.290–0.882 (Figure 3). The sensitivity to change ranged between small ES = 0.005–0.434 for ML perturbations and between small to medium ES = 0.028–0.520 for AP perturbations (Figure 3). Concurrent validity with the recovery performance based on trunk velocities ranged from weak to very strong *r* = 0.09–0.938 for ML perturbations and from weak to strong *r* = 0.009–0.775 for AP perturbations (Figure 3).

No single QRP method was superior to the other calculations. See Supplementary Table 1 for full details.

## Discussion

We developed and evaluated a new method of quantifying recovery performance after treadmill-based gait perturbations using center of pressure data (CoP) obtained from a treadmill-embedded force plate. We compared various implementations of the QRP, with respect to test-retest reliability, sensitivity to change and concurrent validity. Results showed a wide range across these implementations for reliability, sensitivity, and concurrent validity, suggesting that no option is superior and that a choice between these implementations must be made dependent on the constraints and demands of the setting in which the QRP will be used. Theoretically, when evaluating a perturbation protocol only using decelerations of the belt, a QRP based on a non-normalized pre-perturbation episode of 5 s combining AP and ML dimension as input would provide substantial reliability (ICC = 0.855), medium sensitivity to change (ES = −0.496) and moderate validity (*r* =-0.476). For a ML perturbation protocol, a QRP based on a normalized unperturbed walking trial with only the AP dimension as input would provide high reliability (ICC = 0.935) with small sensitivity to change (ES = −0.434), which is still the largest effect size across all options for ML perturbations, and strong validity (*r* = −0.854).

The change from the normal walking pattern can be quantified using Pearson's correlation coefficient or using an area under the curve describing the difference in the time series of the CoP patterns between perturbed and unperturbed gait as a measure of deviation. Both options provide similar results for test-retest reliability and sensitivity to change across all options. In general, the correlation-based option yielded less variable results compared to the deviation-based options, especially for concurrent validity, suggesting correlation-based calculations of the QRP to be more consistent across different perturbations.

Our pilot study suggested that the change from the normal walking pattern was larger in the AP compared to the ML dimension, when perturbations were applied in AP direction and vice versa for perturbations in ML direction. However, similar performance of the QRP was found when using CoP data from either the AP or ML dimension or when AP and ML dimensions were combined. When using the QRP in a setting where perturbations in AP and ML direction are applied, then a correlation-based option using combined AP and ML CoP data provides more reliable and sensitive results for both AP and ML perturbations compared to a deviation-based option combining AP and ML CoP data. Further, when combining CoP data from the AP and ML dimension, the recovery reaction can be captured more completely yielding a more comprehensive analysis of the recovery performance.

For clinical practice, the QRP should preferably not rely on detection of gait events, as automatic detection of gait events from a COP trajectory may not always produce reliable results, due to the large variability in reactive stepping response. Multiple recovery strategies have been observed for trips (Eng et al., 1994) and slips (Yang et al., 2008) and some subjects perform cross-over steps (Vlutters et al., 2016) and backward steps (Yang et al., 2014). In the present study, we manually checked for false or missing gait events. In a clinical setting, this may not be possible and the gait event detection algorithm needs to detect gait events accurately, which may be limited due to the manifold stepping responses after a perturbation. Test-retest reliability and sensitivity to change of QRP based on a gait episode consisting of 5 s were comparable to those of QRP based on a gait episode consisting of three gait cycles, although concurrent validity was lower, when using time-based episodes. Moreover, as detection of gait events is required to calculate spatial-temporal gait parameters such as step length and step time, such measures that use the time or steps required to recover to baseline values of spatial-temporal parameters (Krasovsky et al., 2012) are less suitable for clinical practice than the QRP measures we proposed.

Given the natural variation in walking behavior, segmenting data into gait cycles almost always results in gait cycles of different duration. Therefore, time-normalization is commonly used for comparison of gait patterns. Similarly, during recovery performance, time normalization of the gait cycles may improve the comparison of perturbed and unperturbed gait cycles. However, it may unnaturally stretch gait cycles in case of false or missing gait events. In the current study we corrected false or missing gait events and our data suggests that normalizing the data provides comparable performance for test-retest reliability and sensitivity to change, but increases variability for concurrent validity results compared to non-normalized data.

If no separate reference trial is available, correlation-based QRP measures provide more reliable outcomes than deviation-based measures. However, using a normalized separate reference trial provides the highest concurrent validity across all options. This may be because the same choice was made for the reference measure based on trunk velocities (Rieger et al., 2020). A reference containing three pre-perturbation gait cycles provide similar performance on reliability, sensitivity and validity than a separate reference trial and both options have higher concurrent validity compared to a 5 s pre-perturbation time window as reference. With respect to the length of the time window analyzed, we have previously shown that recovery of the trunk kinematics is achieved within three gait cycles after perturbations of a magnitude such as applied here. This suggests that a pre- and post-perturbation episode of three cycles would be sufficient (Rieger et al., 2020). However, this depends on reliable automatic gait event detection and as mentioned before, this may limit this option.

In clinical practice, online feedback of recovery performance to the therapist is key for monitoring and adjusting perturbation difficulty within a training session. It provides the therapist with objective recovery performance for each perturbation as is preferable over subjective visual judgement. This is possible when the measurement uses pre-perturbed walking as reference. As an alternative, the use of data from a separate reference trial is possible and the advantage may be that anticipation to perturbations does not affect the reference COP pattern. In addition, more gait cycles can be recorded, resulting in a more precise average gait cycle. However, such a trial needs to be taken in the beginning of a training session and may be affected by a lack of familiarization. Therefore, recording a separate reference trial may be time consuming and may need to be recorded in every training session, as a participant's walking speed may change over sessions. Finally, participants may adapt their gait pattern between two perturbations, which is likely to be different compared to the gait pattern of a separate, unperturbed walking reference trial. This would favor for using three pre-perturbed gait cycles, as this provides similar results as a separate reference trial of unperturbed walking. Moreover, we have previously shown that improvement in recovery performance is independent of adaptive changes in the gait pattern (Rieger et al., 2020).

In the current study we evaluated the CoP based QRP measures against a recovery performance based on trunk velocity deviations obtained with motion capturing. It could be argued that the recovery performance measure based on trunk velocity deviations is not yet accepted as a golden standard for quantifying balance recovery after gait perturbations. Alternatively, the concept of Margins of Stability (MoS) has been used to quantify stability of walking. However, stepping responses after a perturbation are manifold, including jumping, skipping, repositioning of the perturbed foot, various side or cross-over steps (Mccrum et al., 2018) and these are difficult to analyze within this framework. Moreover, the MoS concept is based on the assumption that the body can be modeled as an inverted pendulum. In responses after gait perturbations, this assumption is likely to be violated (Bruijn et al., 2013; Hak et al., 2013) Consequently, gait adaptations after experiencing a perturbation, e.g., walking with flexed knees to lower the CoM, may limit the applicability of the MoS (Hof et al., 2005). Therefore, measures based on the deviation in trunk velocities from unperturbed walking provide high test-retest reliability and sensitivity to change and has potential to become the golden standard to quantify recovery after gait perturbations.

### Limitations

In our study, only treadmill belt decelerations were used in the AP direction to provoke backward balance loss and contra-lateral sway perturbations in the ML direction. These perturbations were selected as they are considered the most challenging for each direction eliciting the strongest recovery responses (Roeles et al., 2018; Rieger et al., 2020). However, this implies that information is lacking for perturbations using belt accelerations and ipsilateral sway perturbations. In addition, only one intensity level as perturbation difficulty was used and treadmill speed was fixed for all participants. Further investigation of validity for a variety of perturbation types after more or less challenging perturbations, at various gait speeds and across different target groups are recommended. Finally, we recommend that future recovery performance measures could be based on inertial measurement units (Faber et al., 2009; Miller and Kaufman, 2019) or a simple camera system, such as the Kinect, to capture trunk kinematics (Shani et al., 2017), as this provides more reliable and sensitive outcomes compared to CoP based measures.

For this study we used selected conditions from a previous study (Rieger et al., 2020). For test-retest reliability we selected data exclusively from the control group in that study as some systematic changes we found between time points in the training group. We additionally evaluated reliability over the whole cohort, which confirmed our conclusion that the QRP measure was less reliable than the reference measure based on trunk kinematics. To assess concurrent validity, we used the retention trial over the whole cohort, excluding the pre- and post-intervention trials. Additional evaluation of the effect sizes per group did not lead to a different conclusion. Furthermore, additional analysis of the concurrent validity in the baseline and post-intervention trials did not yield substantially different results.

### Conclusion

Quantifying recovery performance using center of pressure data from a force-plate embedded treadmill device can achieve sufficient reliability and concurrent validity, although less reliable and sensitive to change than trunk velocity measures.

## Data Availability Statement

The raw data supporting the conclusions of this article will be made available by the authors, without undue reservation.

## Ethics Statement

The studies involving human participants were reviewed and approved by Vaste Commissie Wetenschap en Ethiek (VCWE), Vrije Universiteit Amsterdam. The patients/participants provided their written informed consent to participate in this study.

## Author Contributions

MR collected the data and wrote the first draft of the manuscript. MR and JvD analyzed the data. SP, FS, MP, and JvD edited the draft version. All authors contributed to conception and design of the study, manuscript revision, read, and approved the submitted version.

## Conflict of Interest

MR is an early stage researcher, employed by Motek Medical BV and performed the analysis of the data, in collaboration with Vrije Universiteit Amsterdam. Further, SP and FS are employed by Motek Medical BV and a device of this company was used for this experiment. The remaining authors declare that the research was conducted in the absence of any commercial or financial relationships that could be construed as a potential conflict of interest.
