# Supplementary material for: Development of a Balance Recovery Performance Measure for Gait Perturbation Training Based on the Center of Pressure
Source: Front Sports Act Living. 2021 Feb 15;3:617430. doi: 10.3389/fspor.2021.617430 (PMC7917114; doi:10.3389/fspor.2021.617430)
Supplement: Supplementary file 1 [file Data_Sheet_1.PDF]

Table S1: Overview of the results of all QRP implementations against the gold standard trunk velocity deviation.

Measurement	Dimension	Name	Reliability ICC		Sensitivity ES		Validity Pearson's r	
			Sway	Dec	Sway	Dec	Sway	Dec
Deviation (Standard)	AP + ML	Trunk velocity NormTrial	0.897	0.855	0.977	1.028	x	x
Correlation	AP	COP NonNorm Pre 5sec	0.861	0.805	0.015	-0.428	-0.437	-0.497
		COP NonNorm Pre 3cycles	0.813	0.830	0.037	-0.429	-0.346	-0.479
		COP Norm Pre 5sec	0.927	0.697	-0.146	-0.507	-0.561	-0.400
		COP Norm Pre 3cycles	0.934	0.743	-0.162	-0.408	-0.849	-0.614
		COP NonNorm Trial	0.613	0.675	-0.304	-0.384	-0.276	-0.507
		COP Norm Trial	0.935	0.743	-0.434	-0.516	-0.854	-0.633
Correlation	ML	COP NonNorm Pre 5sec	0.801	0.882	-0.192	-0.520	-0.385	-0.455
		COP NonNorm Pre 3cycles	0.835	0.857	-0.187	-0.494	-0.407	-0.424
		COP Norm Pre 5sec	0.840	0.771	-0.105	-0.451	-0.394	-0.475
		COP Norm Pre 3cycles	0.921	0.586	-0.118	-0.146	-0.152	-0.524
		COP NonNorm Trial	0.828	0.751	-0.291	-0.386	-0.383	-0.465
		COP Norm Trial	0.919	0.590	-0.160	-0.210	-0.154	-0.527
Correlation	AP + ML	COP NonNorm Pre 5sec	0.862	0.855	-0.100	-0.496	-0.501	-0.475
		COP NonNorm Pre 3cycles	0.848	0.867	-0.085	-0.492	-0.448	-0.451
		COP Norm Pre 5sec	0.904	0.766	-0.160	-0.497	-0.598	-0.464
		COP Norm Pre 3cycles	0.916	0.717	-0.165	-0.248	-0.817	-0.665
		COP NonNorm Trial	0.719	0.724	-0.360	-0.415	-0.363	-0.495
		COP Norm Trial	0.905	0.723	-0.322	-0.362	-0.821	-0.681

Deviation	AP	COP NonNorm Pre 5sec	0.596	0.290	-0.190	-0.248	0.090	-0.198
		COP NonNorm Pre 3cycles	0.678	0.624	-0.022	-0.030	0.408	0.017
		COP Norm Pre 5sec	0.502	0.448	-0.168	-0.097	0.090	0.009
		COP Norm Pre 3cycles	0.907	0.768	0.005	0.028	0.900	0.534
		COP NonNorm Trial	0.821	0.682	0.339	0.256	0.527	0.170
		COP Norm Trial	0.921	0.834	0.406	0.399	0.938	0.704
Deviation	ML	COP NonNorm Pre 5sec	0.745	0.613	0.201	0.214	0.140	0.121
		COP NonNorm Pre 3cycles	0.725	0.871	0.216	0.201	0.697	0.416
		COP Norm Pre 5sec	0.827	0.657	0.207	0.187	0.085	0.182
		COP Norm Pre 3cycles	0.486	0.591	0.138	0.241	0.795	0.660
		COP NonNorm Trial	0.819	0.777	0.243	0.313	0.713	0.420
		COP Norm Trial	0.799	0.744	0.198	0.257	0.885	0.731
Deviation	AP + ML	COP NonNorm Pre 5sec	0.696	0.669	0.115	0.141	0.155	0.021
		COP NonNorm Pre 3cycles	0.765	0.862	0.165	0.159	0.689	0.345
		COP Norm Pre 5sec	0.823	0.568	0.122	0.134	0.111	0.146
		COP Norm Pre 3cycles	0.759	0.841	0.112	0.208	0.869	0.641
		COP NonNorm Trial	0.863	0.793	0.276	0.312	0.732	0.412
		COP Norm Trial	0.876	0.859	0.250	0.287	0.930	0.775
